# Supplementary material for: Exploring cellular changes in ruptured human quadriceps tendons at single‐cell resolution
Source: J Physiol. 2025 Apr 15;603(16):4535–54. doi: 10.1113/JP287812 (PMC12369293; doi:10.1113/JP287812)
Supplement: Supplementary file 2 — Supplementary Figure S1 Supplementary Figure S2 Supplementary Figure S3 Supplementary Figure S4 Supplementary Figure S5 Supplementary Figure S6 Supplementary Figure S7 Supplementary Figure S8 Supplementary Figure S9 Supplementary Figure S10 Supplementary Figure S11 [file TJP-603-4535-s001.docx]

**Supplementary Information**

**Exploring cellular changes in ruptured human quadriceps tendons at single-cell resolution**

Jolet Y. Mimpen^1,2^*^#^, Mathew J. Baldwin^1^*, Claudia Paul^1^, Lorenzo Ramos-Mucci^1^, Alina Kurjan^1^, Carla J. Cohen^1,3^, Shreeya Sharma^1^, Marie S.N. Chevalier Florquin^4^, Philippa A. Hulley^1^, John McMaster^5^, Andrew Titchener^5^, Alexander Martin^5^, Matthew L. Costa^1,5^, Stephen E. Gwilym^1,5^, Adam P. Cribbs^1,6^, Sarah J.B. Snelling^1^

* Jolet Y. Mimpen and Mathew J. Baldwin contributed equally

*^1^ The Botnar Institute of Musculoskeletal Sciences, Nuffield Department of Orthopaedics Rheumatology and Musculoskeletal Sciences, University of Oxford, Oxford, United Kingdom*

*^2^ Kennedy Institute of Rheumatology, Nuffield Department of Orthopaedics Rheumatology and Musculoskeletal Sciences, University of Oxford, Oxford, United Kingdom*

*^3^ Centre for Computational Biology, MRC Weatherall Institute of Molecular Medicine, University of Oxford, Oxford, UK*

*^4^ Leiden University Medical Center, Leiden University, Leiden, the Netherlands*

*^5^ Oxford University Hospital NHS Foundation Trust, Oxford, UK*

*^6^ Oxford Centre for Translational Myeloma Research University of Oxford, Oxford, UK*

**^#^ Correspondence:**

Dr Jolet Mimpen

Nuffield Department of Orthopaedics Rheumatology and Musculoskeletal Sciences

United Kingdom

[jolet.mimpen@ndorms.ox.ac.uk](mailto:jolet.mimpen@ndorms.ox.ac.uk)

ORCID: 0000-0003-4464-242X


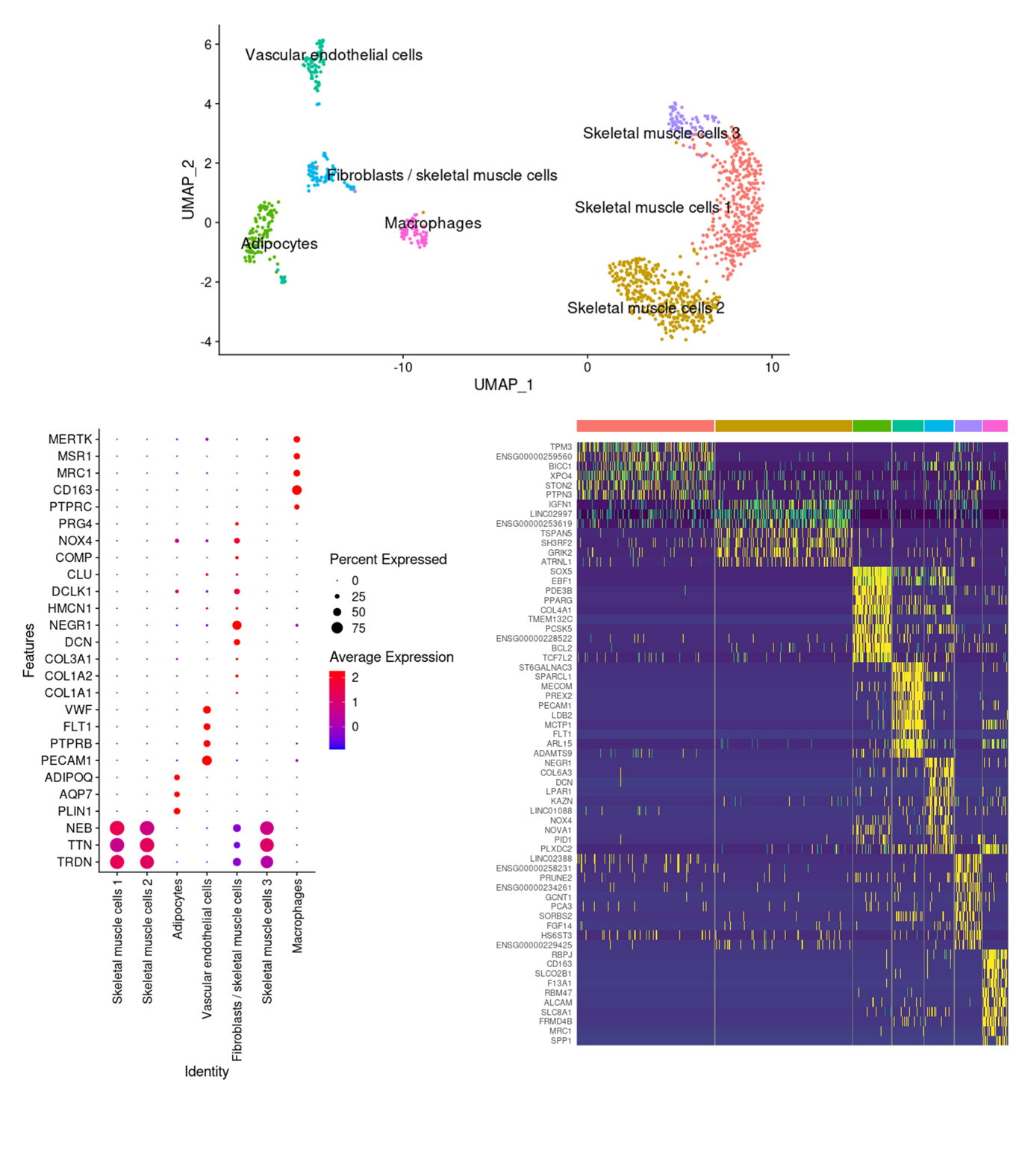


**Supplementary Figure 1.** Cell clusters and gene expression of canonical markers in data from patient MSK1250.


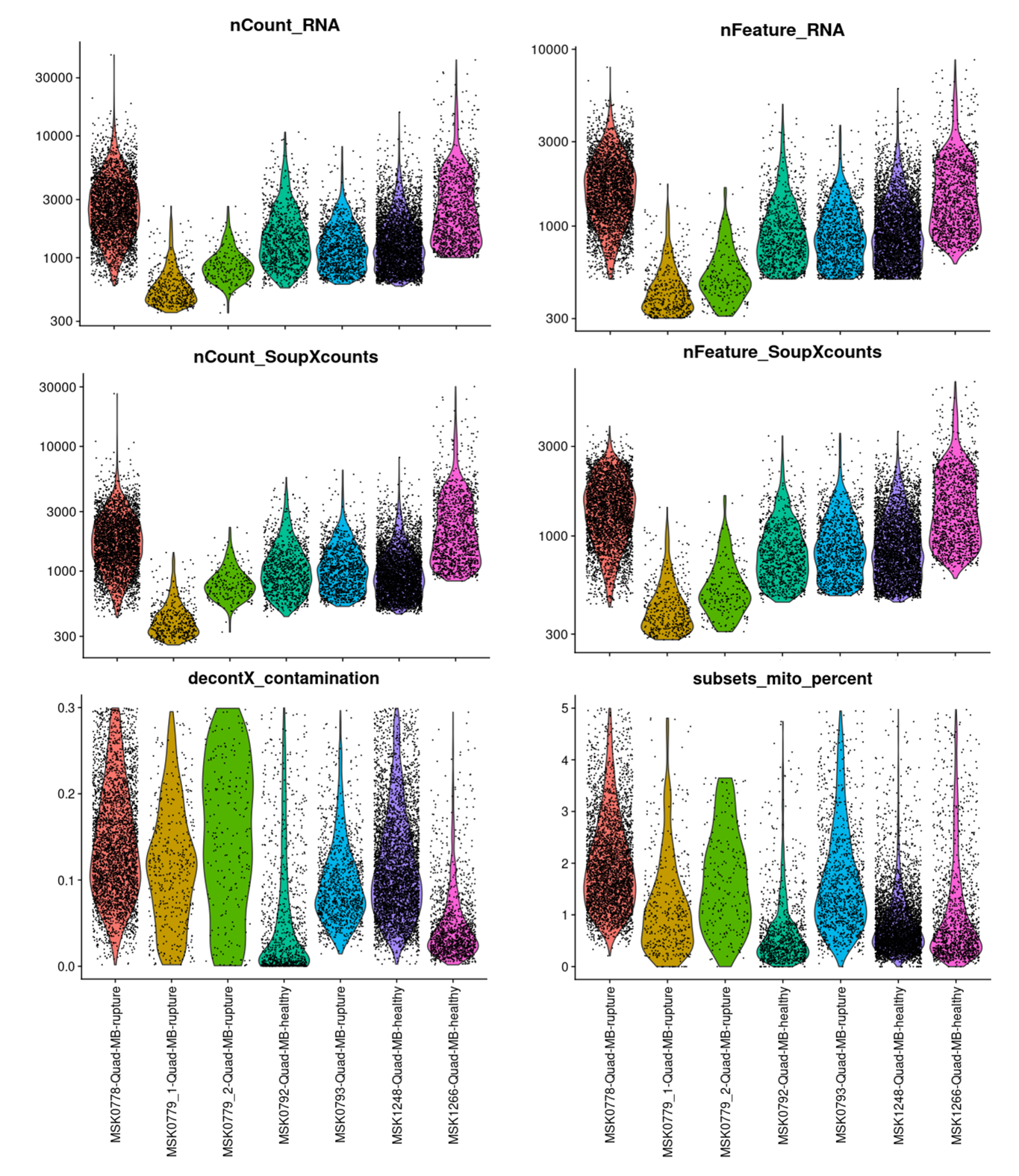


**Supplementary Figure 2**. QC metrics of each dataset, including number of counts (nCount) and number of features (nFeature) in the RNA and SoupXcounts assays, decontXcontamination score, and percentage of mitochondrial reads (subsets_mito_percent).

**
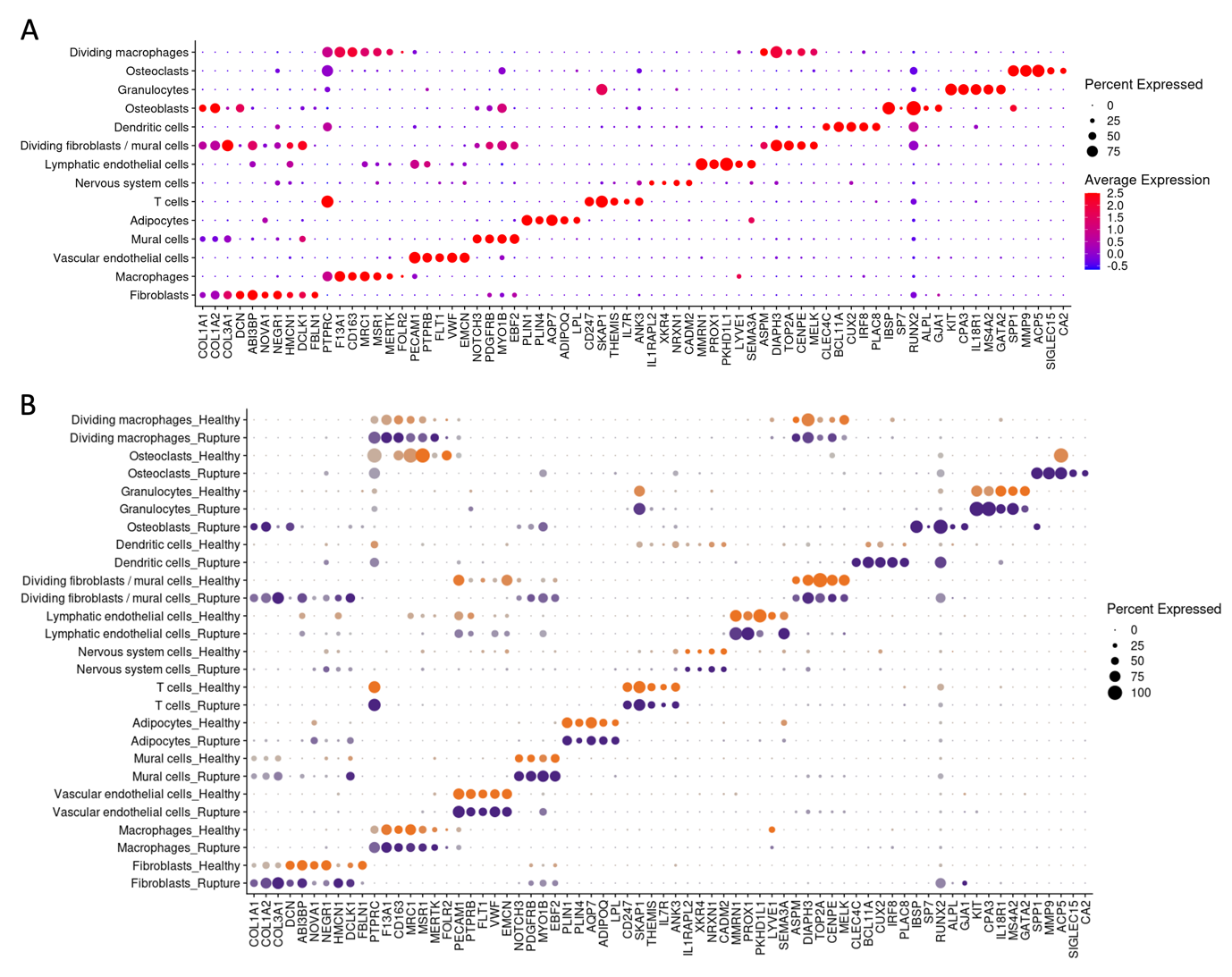
Supplementary Figure 3.** Expression of canonical markers in each identified cell subset, either overall (A) or split by healthy (purple) and rupture (orange).


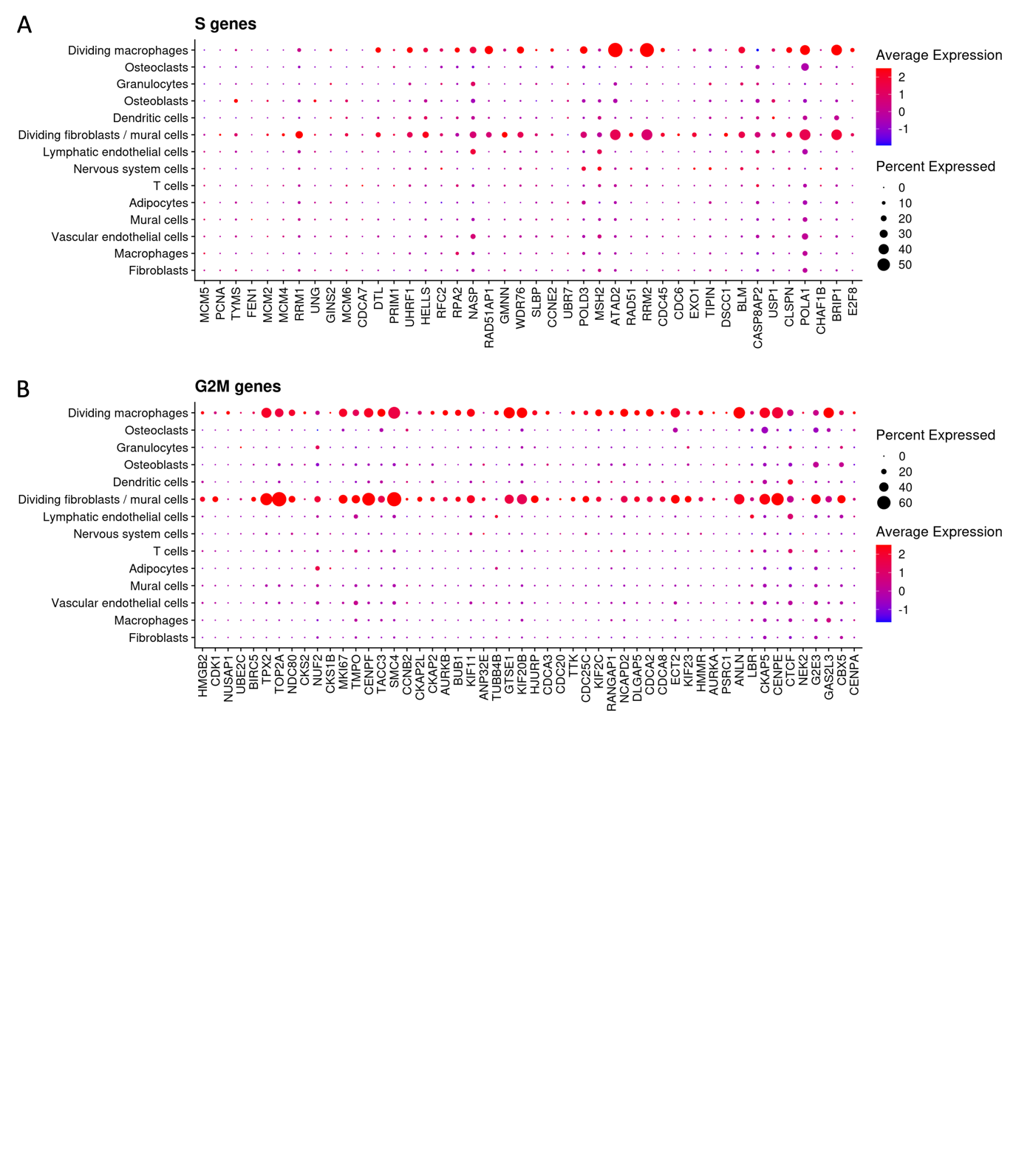
**Supplementary Figure 4**. Expression of S- and G2M-phase genes in each cell subset as presented in Figure 1.


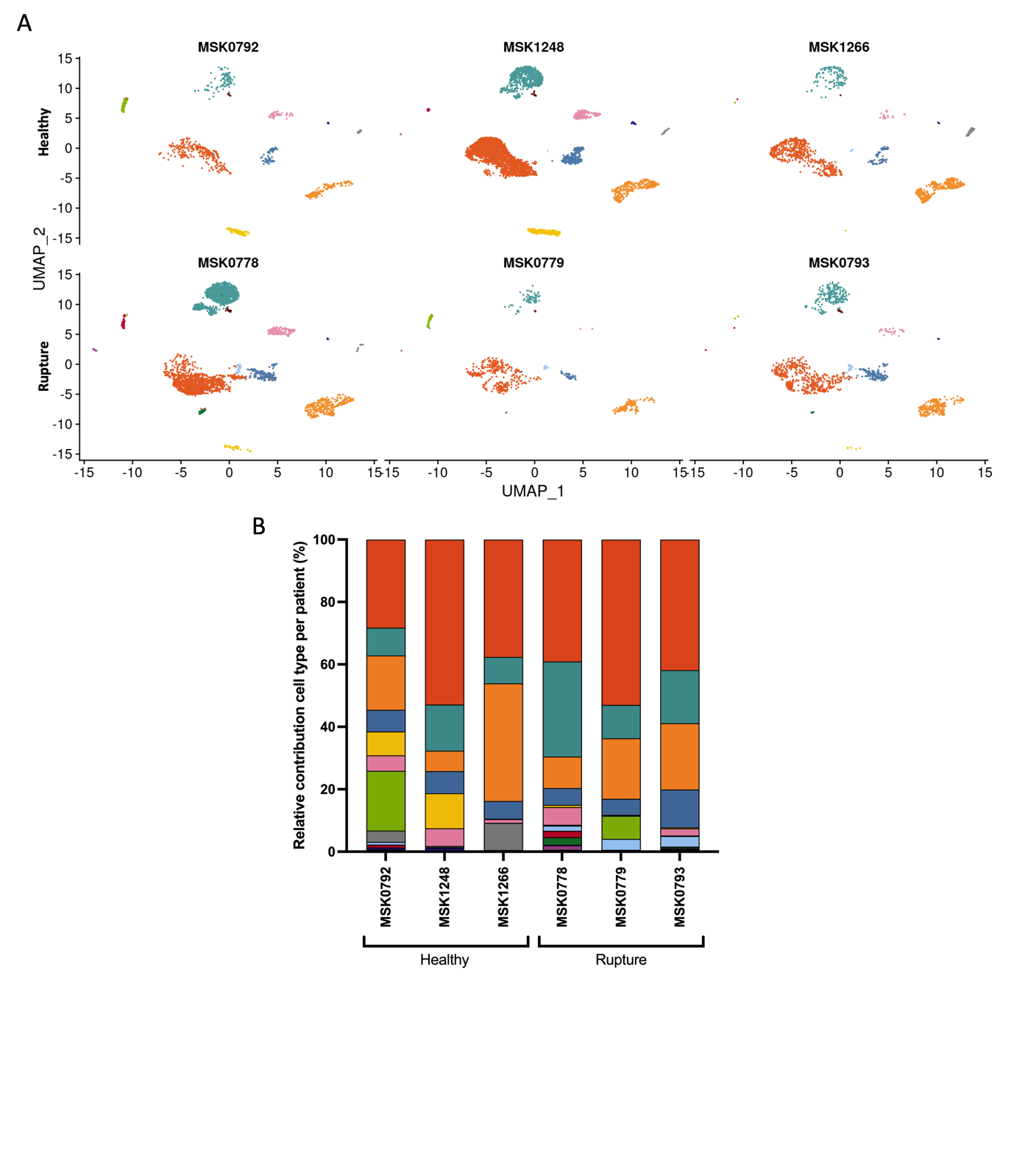


**Supplementary Figure 5**. Single nucleus RNA sequencing results by disease status and patient. (A) UMAP embedding of 12,817 nuclei from quadriceps tendons split by patient; top row are healthy donors and bottom row are donors of ruptured quadriceps samples. (B) Mean cell type frequency per patient.


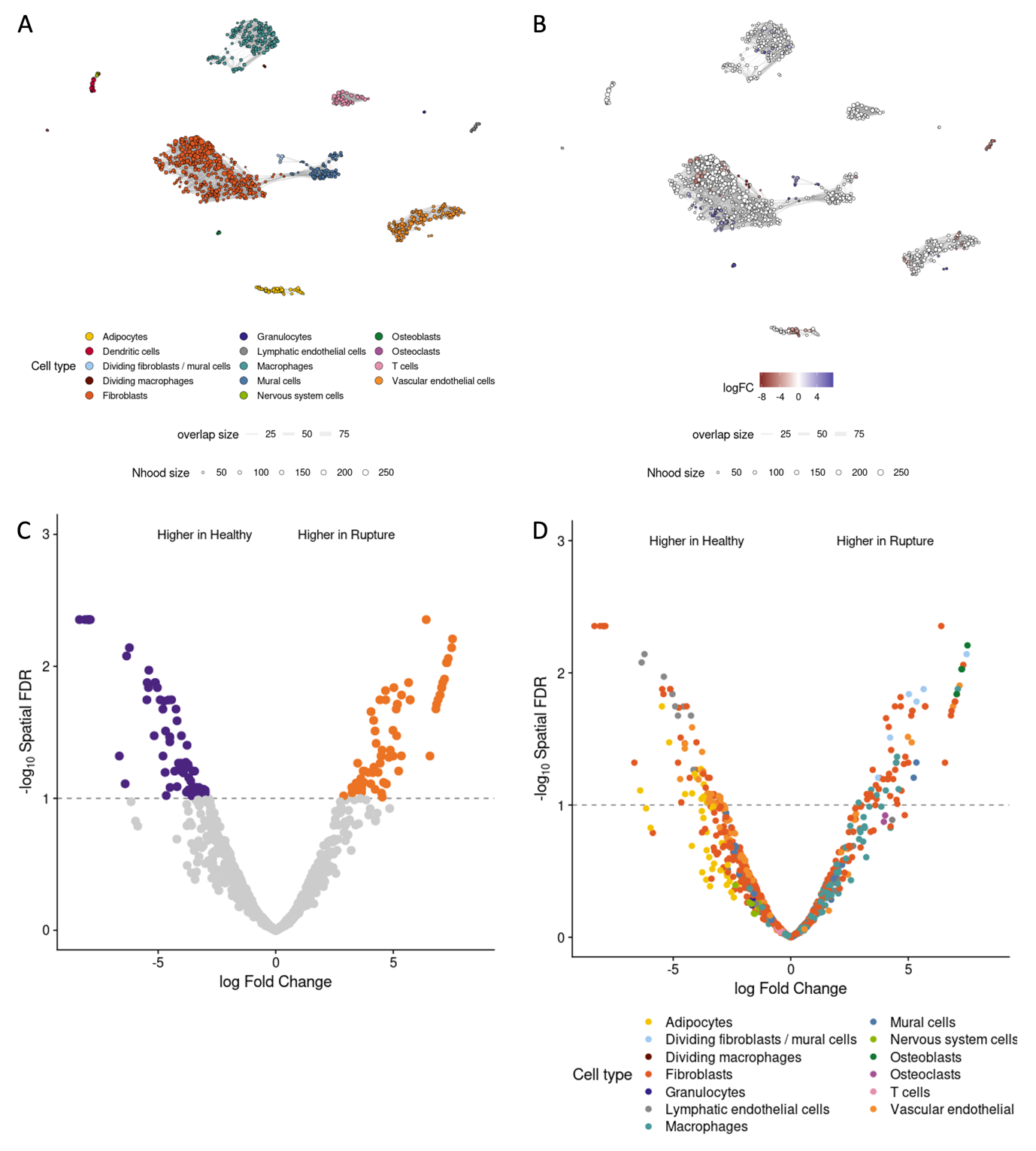


**Supplementary Figure 6**. MiloR differential abundance analysis. MiloR was used to assign neighbourhoods (A) and test these neighbourhoods for differential abundance (B). (C-D) Volcano plots depict the differential abundance of each neighbourhood, either coloured by (C) tendon disease (ruptures (orange) or healthy (purple)), or by (D) cell type.


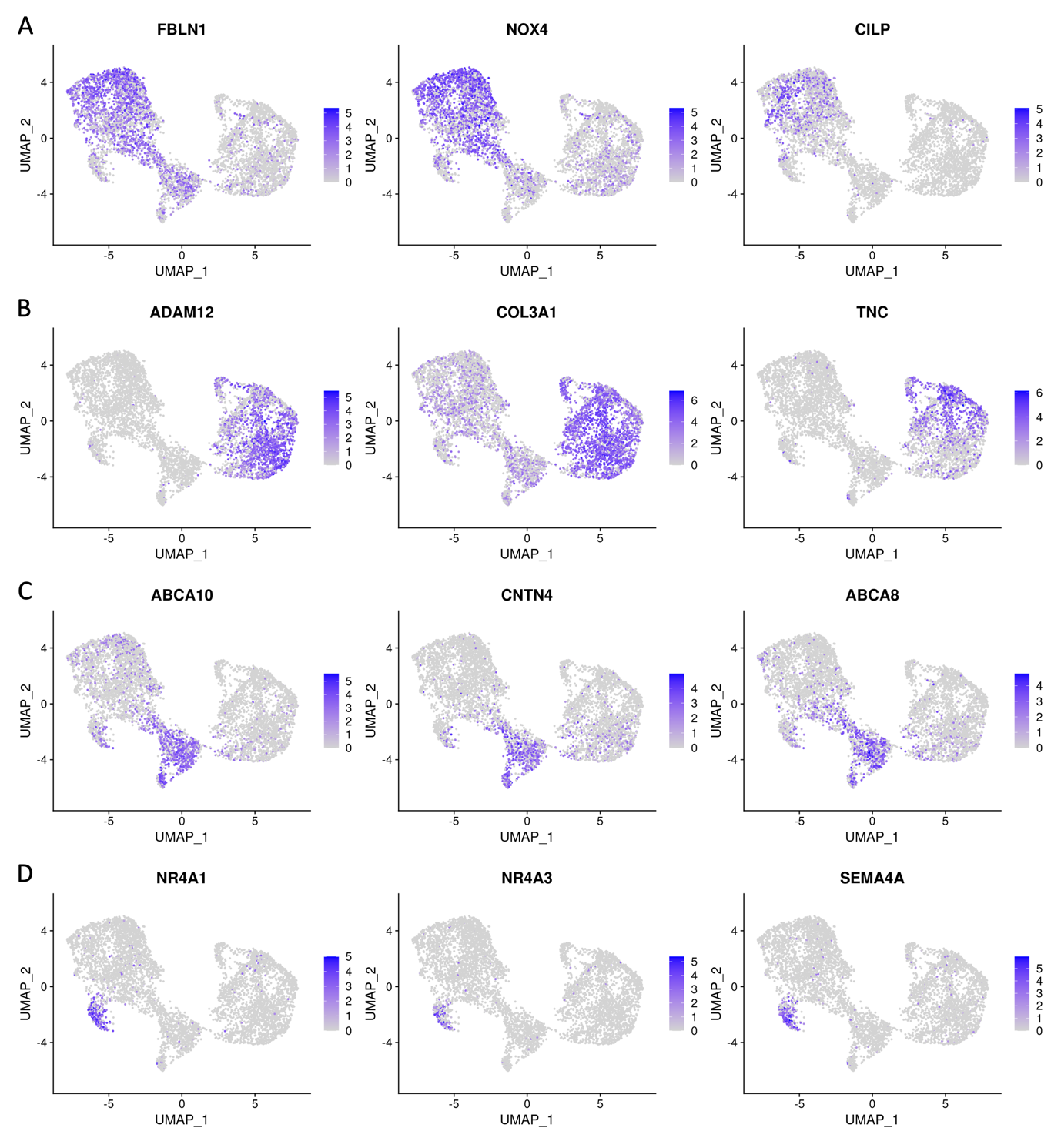


**Supplementary Figure 7**. FeaturePlots of three highly differentially expressed genes in each fibroblast subcluster (as shown in Figure 3D), namely FBLN1^hi^ fibroblasts (A), ADAM12^hi^ fibroblasts (B), ABCA10^hi^ fibroblasts (C), and NR4A1^hi^ fibroblasts (D).


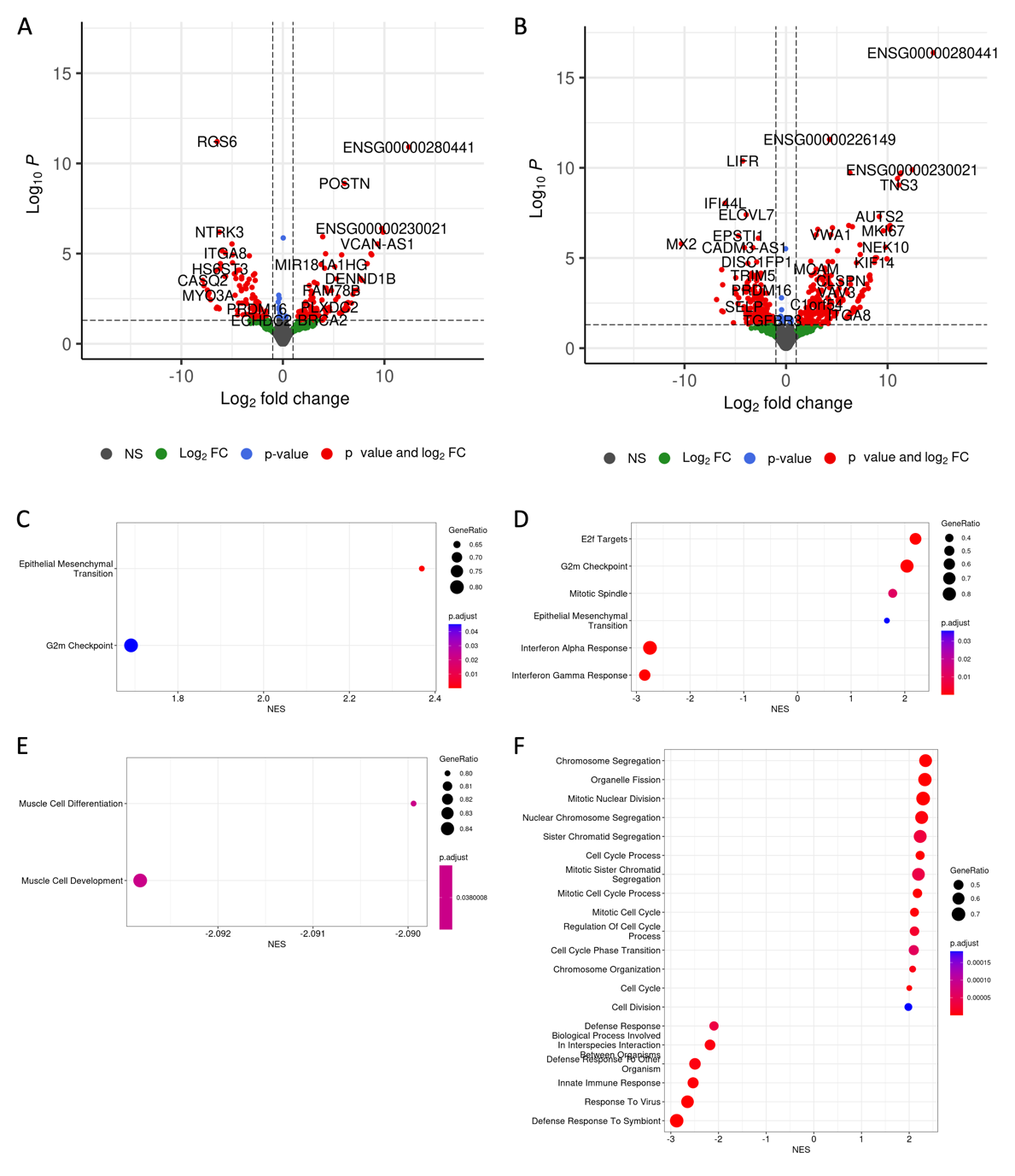


**Supplementary Figure 8**. Differential gene expression results of mural cells (left) and vascular endothelial cells (right), including volcanoplots (A-B), Hallmark pathway results (C-D), and Gene Ontology Biological Processes results (E-F), either all (E) or top 20 (F).

**
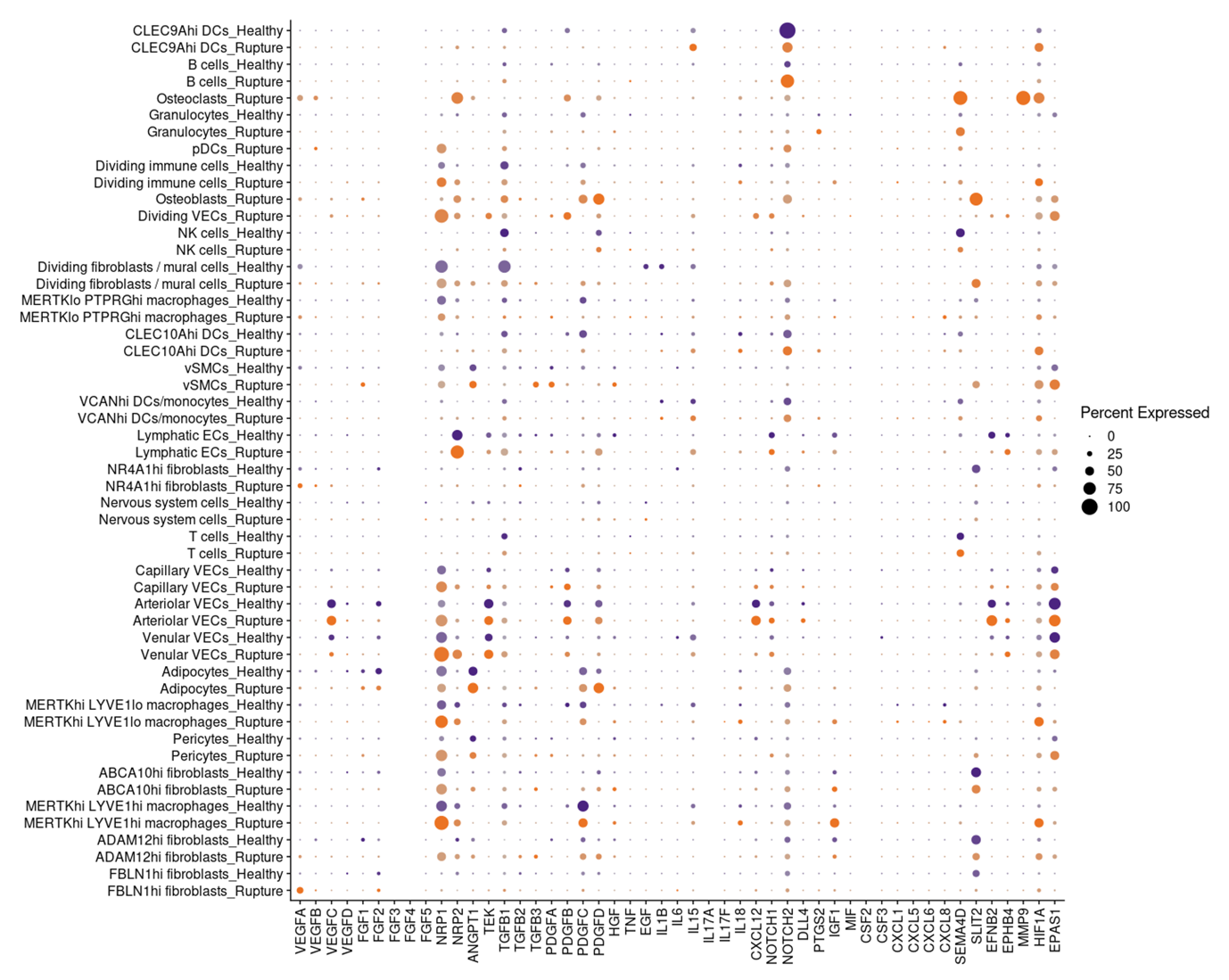
Supplementary Figure 9**. The expression of pro-angiogenic factors by all the identified clusters, split by healthy (purple) and rupture (orange).


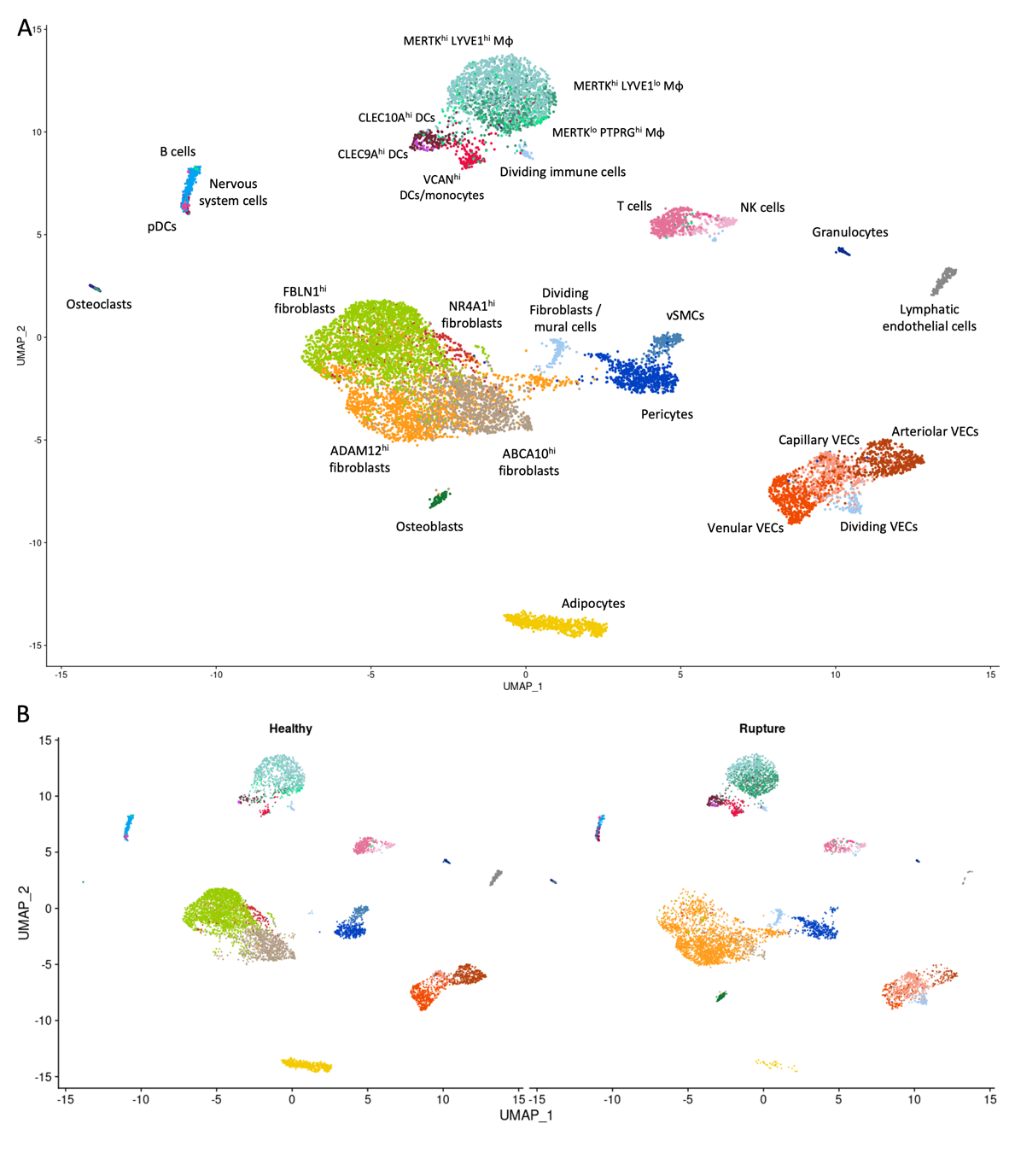


**Supplementary Figure 10**. UMAP embedding with updated cell annotation based on the subclustered fibroblast, endothelial, and immune cell types, either overall (A) or split by tendon disease (B). DC = dendritic cells, MΦ = macrophages, VEC = vascular endothelial cells, vSMCs = vascular smooth muscle cells.


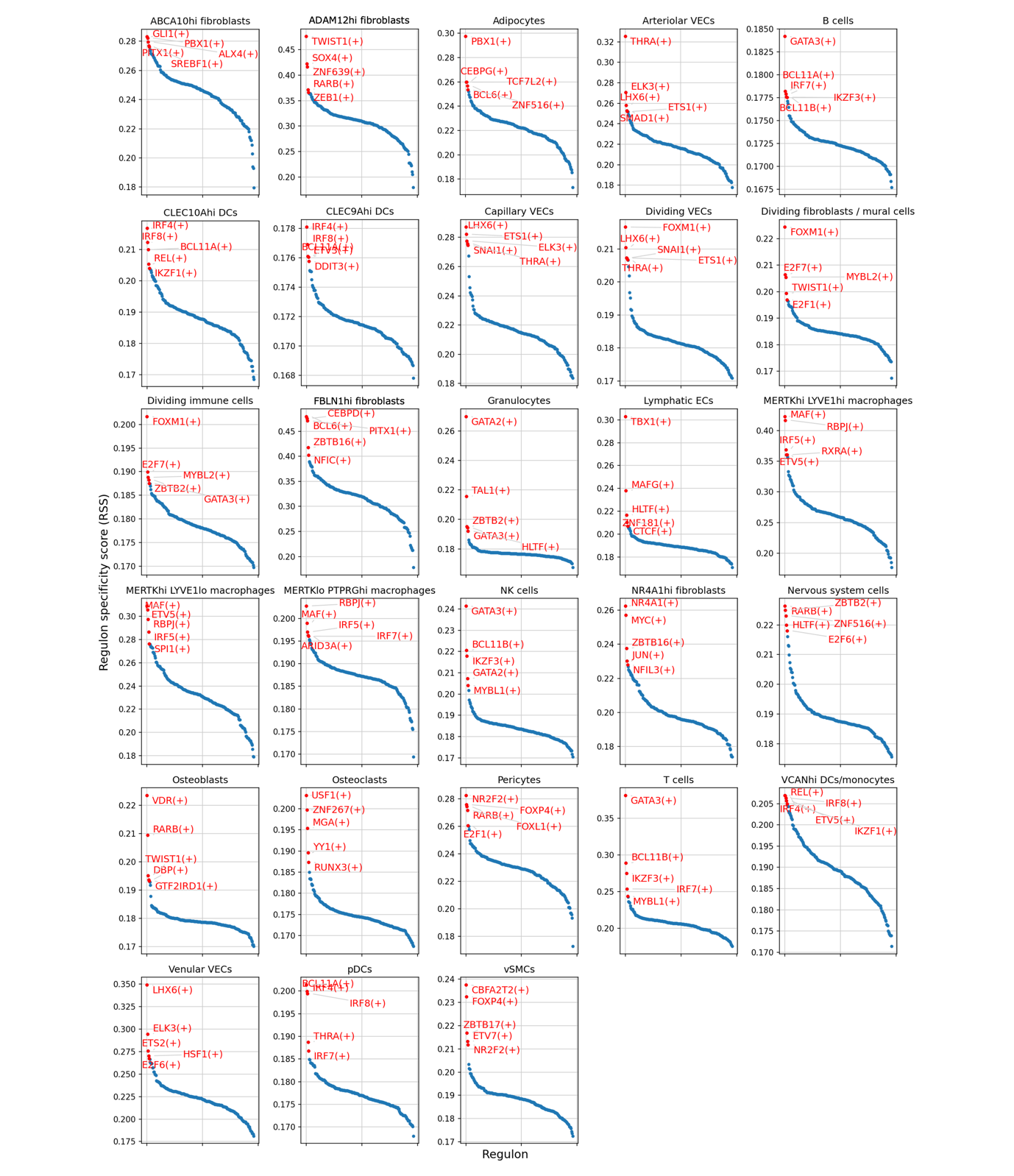


**Supplementary Figure 11**. Regulon specificity scores (RSS) for each of the identified cluster. For each cluster, the top 5 regulons are highlighted in red.
